# Triptolide in renal disorders: dual roles of therapeutic potential and nephrotoxicity: a narrative review

**DOI:** 10.1080/13880209.2026.2616270

**Published:** 2026-01-21

**Authors:** Lan Yan, Lulu Zhang, Xiaomeng Zhang, Changqi Shi, Qi Geng, Lin Lin, Ning Zhao, Li Li, Xiaojuan He, Yong Tan, Xinyu Ji, Cheng Lu

**Affiliations:** Institute of Basic Research in Clinical Medicine, China Academy of Chinese Medical Sciences, Beijing, China

**Keywords:** Triptolide, kidney disease, inflammation, oxidative stress, cell death

## Abstract

**Background:**

Triptolide (TP), derived from *Tripterygium wilfordii*, exhibits anti-inflammatory, immunosuppressive, and antifibrotic properties with potential for treating renal diseases, but its clinical use is restricted by dose-dependent nephrotoxicity.

**Objective:**

The aim of this review is to comprehensively summarize the dual roles of TP, elucidate its therapeutic mechanisms and nephrotoxic pathways, and to explore strategies to mitigate its toxicity.

**Methods:**

A literature search was performed using the PubMed and Web of Science databases. The search covered publications from the earliest available date until November 2025. The key search terms included ‘triptolide’, ‘renal’, ‘kidney’ and their combinations.

**Results:**

TP exerts dose-dependent dual effects in renal models. Therapeutic doses (typically ≤200 μg/kg *in vivo*) demonstrate efficacy in modulating immune responses, protecting podocytes, promoting apoptosis in hyperproliferative cells and inhibiting renal fibrosis. Conversely, its nephrotoxicity manifests at supratherapeutic doses (often >400 μg/kg *in vivo*) through oxidative stress, inflammation, metabolic dysregulation, and direct damage to renal tubular cells. The therapeutic efficacy and toxicity of TP are critically contingent on both dose and temporal parameters.

**Conclusion:**

TP holds significant but challenging potential for renal therapy. Future research should define its therapeutic window and advance strategies such as structural analogs, targeted delivery systems, and combination therapies to effectively separate efficacy from toxicity for clinical translation.

## Introduction

*Tripterygium wilfordii Hook F (Celastraceae)* (TwHF) is a widely used natural medicine in clinical practice and is often administered to treat autoimmune and inflammation-related diseases (Luo et al. 2019; Zhang et al. 2024). Triptolide (TP), an active component extracted from TwHF, has potent therapeutic effects on the regulation of immune dysfunction and the inhibition of severe inflammatory responses, and is considered as one of the most promising natural compounds for drug development (Corson and Crews 2007; Cai et al. 2023). Recent advances in research have revealed that TP possesses diverse pharmacological properties, including anti-inflammatory, immunosuppressive, cell proliferation and death regulatory, antifibrotic, and antitumor activities, indicating its potential efficacy in kidney diseases (Liang et al. 2021; Wang et al. 2022).

Kidney diseases affect more than 850 million people worldwide, and pose a serious threat to global health (Jager et al. 2019). The pathogenesis of various kidney disorders commonly involves dysfunction or injury to key renal cells, including podocytes, renal tubular epithelial cells (RTECs), and mesangial cells (MCs) (Fissell and Miner 2018). Such injuries disrupt the glomerular filtration barrier, provoke inflammatory responses, and promote fibrosis, ultimately leading to proteinuria and a decline in renal function (Ostermann et al. 2020; Ramya Ranjan Nayak et al. 2023).

This review synthesizes evidence supporting the role of TP in maintaining renal structure and cellular physiology, including the preservation of GFB integrity, protection of podocytes, and regulation of MCs proliferation to reduce proteinuria. Notably, TP has a narrow therapeutic window: its effective doses for treating kidney diseases are close to nephrotoxic thresholds, and it induces nephrotoxicity in both normal and pathological models. Thus, this review provides a comprehensive analysis of the effects of different doses, administration durations, and delivery modes of TP on renal diseases. It also summarizes strategies to enhance the efficacy of TP and mitigate its toxicity, aiming to offer insights into its safe and effective application in renal disorders.

## Method

The full electronic search strategies were as follow: PubMed: (‘triptolide’) AND (‘kidney’ OR ‘renal’); Web of Science: TS=(‘triptolide’) AND TS=(‘renal’ OR ‘kidney’). The search was conducted from the inception of each database until November 2025.

The inclusion criteria were as follows: (1) original article *in vivo* or *in vitro*; (2) investigations into the therapeutic mechanisms and/or nephrotoxic mechanisms of TP; (3) studies focused on the renal system or renal cells. The exclusion criteria were as follows: (1) review articles, case reports, meta-analyses, and commentaries; (2) non-English literature; (3) studies not related to the renal system; (4) articles for which the full text could not be retrieved or contained invalid data.

**Figure 1.**
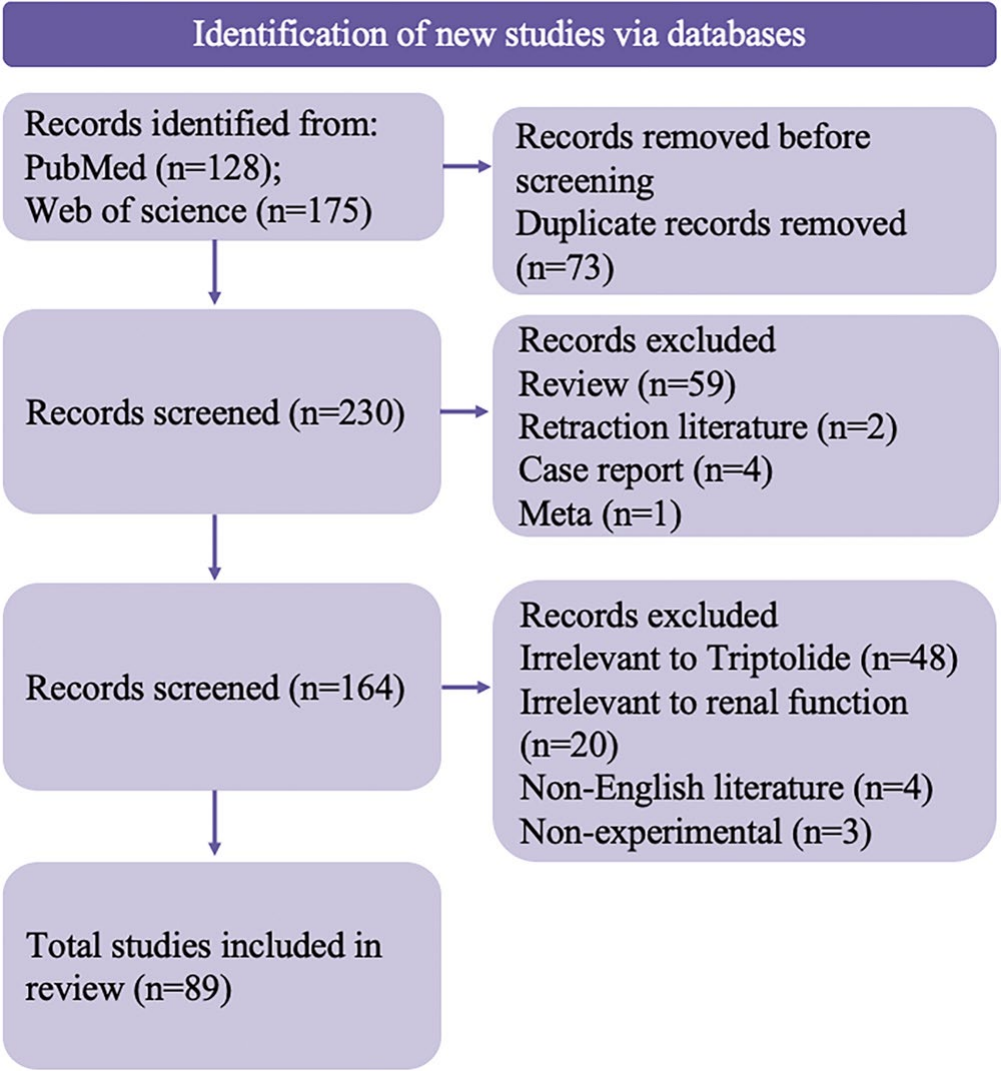
Flow diagram of the study identification, screening, and inclusion process.

A total of 303 records were identified through database searching (PubMed, *n* = 128; Web of Science, *n* = 175). After 73 duplicates were removed, 230 records were screened. Based on title and abstract, 59 records were excluded for review (*n* = 59), 2 records were excluded for retraction literature (*n* = 2), 4 records were excluded for case report (*n* = 4), 1 record were excluded for meta (*n* = 1). Based on full paper, 48 records were excluded for being irrelevant to triptolide (*n* = 48), irrelevant to the renal system (*n* = 20), non-English (*n* = 4), or nonexperimental studies (*n* = 3). The full texts of the remaining 164 articles were assessed for eligibility, of which 89 studies met all the inclusion criteria and were included in the review (Figure 1).

## Impact of triptolide on kidney cells

### Effect of triptolide on renal tubular epithelial cells

RTECs are central targets in kidney injury. Upon damage, they undergo maladaptive phenotypic shifts, driving disease progression through the release of proinflammatory and profibrotic mediators (Kirita et al. 2020). Specifically, damaged RTECs release proinflammatory factors such as IL-1β, tumor necrosis factor-alpha (TNF-α), colony-stimulating factor-1 (CSF-1), and Fas ligand. These factors activate extracellular signal-regulated kinase 1/2, p38 mitogen-activated protein kinase (MAPK), nuclear factor-κB (NF-κB), signal transducer and activator of transcription (STAT) signaling, and TGF-β signaling. They also secrete multiple chemokines like C-X-C motif chemokine ligand 10 (CXCL10) and C-C motif chemokine ligand 2 (CCL2), and generate a large amounts of reactive oxygen species (ROS) to promote oxidative stress, thus triggering interstitial inflammation. Conversely, damaged RTECs promote the expression of TGF-β, Wnt, and Notch factors, driving renal fibrosis (Liu et al. 2018).

RTECs serve as critical target cells in multiple kidney diseases, with their functional state dictating disease outcomes. In acute kidney injury (AKI), RTECs are the primary cell type affected, and their regenerative capacity is pivotal for renal function recovery. Mild AKI typically resolves through adaptive repair mechanisms initiated by RTECs, whereas severe or persistent injury can trigger pathological responses—including abnormal adaptive repair, phenotypic transformation, and growth arrest—that ultimately lead to renal fibrosis (Li et al. 2024). In contrast, uncontrolled RTEC proliferation is a hallmark of autosomal dominant polycystic kidney disease (ADPKD), where it drives cyst formation and disease progression. Thus, RTECs exhibit context-dependent cell cycle dysregulation: growth arrest in AKI versus excessive proliferation in ADPKD, highlighting their central role in diverse patterns of kidney injury.

#### Triptolide protects renal tubular epithelial cells from abnormal proliferation

A key pathogenic mechanism in kidney diseases involves cellular dysregulation arising from an imbalance between renal parenchymal cell death and the maladaptive proliferation or recruitment of cells (Sanz et al. 2023). For instance, in acquired cystic kidney disease and renal tumors, abnormally proliferating cells—derived from RTECs—exhibit a higher proliferation rate than their death rate, driving disease progression (Lee et al. 2021; Boussios et al. 2023).

In ADPKD, mutations in PKD1 or PKD2 lead to the aberrant activation of proliferative signaling pathways, such as the JAK/STAT pathway, driving renal tubular epithelial cell proliferation and cyst formation (Bergmann et al. 2018). Accumulating evidence indicates that TP promotes cell death in patients with PKD and reduces the burden of renal cysts. In a clinical trial, PKD patients with proteinuria treated with preparations of TP for six months experienced a significant reduction in proteinuria, inhibited growth of renal cysts, and stable renal function (Chen et al. 2014). In a preclinical study using a *Han:SPRD* rats model and WT9-12 cells, found that TP inhibited the JAK2-STAT3 signaling pathway phosphorylation by suppressing IL-6, reducing proliferating cell nuclear antigen (PCNA) expression and increasing apoptosis (Jing et al. 2018). TP also modulates Ca^2+^ release in a PC2-dependent manner. Treatment with 100 nM TP resulted in higher caspase-3 activity in PC2-expressing RTECs than in PC2-deficient cells. Additionally, TP inhibited the growth of a murine kidney PC1-null cell line (Pkd1^−/−^) by increasing the expression of p21 downstream of PC1 through the JAK/STAT pathway and reduced the cyst burden in the Pkd1−/− mouse model (Leuenroth et al. 2007). Notably, TP has been shown to reduce the cyst burden in the initial, progressive and expansion stages of PKD. Researchers have used the *Pkd1^flox/−^; Ksp-Cre* model, which is characterized by minimal cysts at birth and rapid progression. By intraperitoneally administering TP (250 μg/kg) to lactating mother mice, TP was transmitted through breast milk to the neonatal mice. This intervention reduced the number of cysts and the weight of neonatal mice and diminished the cystic burden by inhibiting Ki-67, suppressing early proliferative phases during cyst initiation and reducing kidney enlargement (Leuenroth et al. 2008). In the *Pkd1flox/flox;Mx1Cre* model, the effects of TP on cyst formation from the neonatal to the adult stages were subsequently evaluated. TP treatment reduced the overall cyst burden and significantly decreased the number of small cysts that formed at the periphery of the renal cortex (Leuenroth et al. 2010).

Renal cell carcinoma (RCC) is a malignant tumor originating from RTECs and accounts for 2-3% of all adult malignancies (Liu et al. 2024). Each year, there are approximately 209,000 new cases and 102,000 deaths occur globally (Young et al. 2024). A member of the TNF family, TNF-associated apoptosis-inducing ligand (TRAIL), exhibits potent antitumor activity with minimal cytotoxicity to normal cells and tissues (von Karstedt et al. 2017). TP acts as a TRAIL sensitizer, enhancing cell death in RCC by increasing the sensitivity of human RCC cells (such as ACHN, A498, Caki-1, 769-P, and 786-O) to TRAIL-induced apoptosis by upregulating the expression of TRAIL-R1, TRAIL-R2, TRAIL-R3, and TRAIL-R4 (Brincks et al. 2015). Furthermore, within the concentration range of 12.5–50 nM, TP exhibits potent pro-apoptotic effects, inducing RCC cells (786-O and OS-RC-2) to progress from the G1 phase to the S phase, while simultaneously preventing cell cycle progression from the S phase. This accumulation of cells in the S phase leads to RCC apoptosis (Li et al. 2011).

Overall, TP exerts potent pro-apoptotic effects in RTECs with abnormal proliferation, making it effective for treating PKD and RCC. In PKD, TP reduces cyst burden and improves renal function through multiple pathways: PC2-dependent Ca^2+^ release, inhibition of the JAK/STAT pathway, and downregulation of proliferation markers (Ki-67, PCNA). In RCC, TP enhances apoptosis by sensitizing cells to TRAIL and inducing S-phase cell cycle arrest.

#### Triptolide protects renal tubular epithelial cells from fibrosis

Renal fibrosis is the central pathological process driving the progression of diabetic kidney disease (DKD) to end-stage renal failure (Hu et al. 2023). A critical event in this process is the activation of injured RTECs, which secrete a persistent stream of profibrotic mediators.

TP counteracts DKD fibrosis through a multitargeted attacks on key signaling cascades. Its most established action is the direct suppression of the canonical TGF-β/Smad pathway. TP downregulates the expression of TGF-β1 and its effector Smad3, upregulates the expression of inhibitory Smad7, and reduces the expression of downstream fibrotic markers such as α-smooth muscle actin (α-SMA), thereby inhibiting extracellular matrix (ECM) production (Pang and Gu 2021). One study used TGF-β1-stimulated NRK-49F cells to focus on the regulation of ECM synthesis by TP and found that 10 ng/ml TP inhibited ECM synthesis (which reduced the levels of collagen type III and fibronectin) by suppressing Smad2 activation (Zhu et al. 2010).

The activation of TGF-β1 can also activate the phosphoinositide 3 kinase (PI3K)-Akt pathway in a Smad-independent manner to promote DKD fibrosis. Phosphatase and tensin homologue (PTEN) is a natural inhibitor of PI3K/Akt signaling and its expression is downregulated in high glucose (HG)- exposed RTECs (Khokhar et al. 2020). In human HK-2 and mouse MCT cells, growth factors such as HG, TGF-β, and IL-6 and the E3 ligase MEX3C catalyze the K27-linked polyubiquitination of PTEN (PTEN^K27-polyUb), converting it into a profibrotic protein that stabilizes epithelial–mesenchymal transition (EMT) transcription factors. Surprisingly, through a sandwich ELISA using His6-TUBE and Ub-PTEN (K80) antibodies to screen and customize a compound library, it was found that TP is an effective inhibitor of MEX3C enzymatic activity, effectively inhibiting PTEN^K27-polyUb^ activity in DKD models, restoring the PTEN’s phosphatase function of PTEN and alleviating EMT (Li et al. 2019).

The antifibrotic efficacy of TP extends to immunomodulation within the tubulointerstitium. Treatment with 10 ng/mL TP suppressed the overexpression of antigen-presenting (class II MHC) and costimulatory (B7-1/B7-2) molecules, decreasing the immunostimulatory capacity of RTECs (Li et al. 2002). Treatment with 4–8 ng/mL TP suppressed TNF-α-induced upregulation of C3, CD40 and B7h (Hong et al. 2002). Treatment with 600 μg/kg TP by daily gavage significantly attenuated tubulointerstitial fibrosis (Yuan et al. 2011).

In summary, TP combats DKD fibrosis through a concerted mechanism, such as direct inhibition of the core TGF-β/Smad and PI3K/Akt pathways and blockade of a novel MEX3C-PTEN ubiquitination axis that drives the EMT and immunomodulation of activated RTECs to reduce interstitial inflammation. These multifaceted actions collectively preserve the renal architecture by reducing ECM deposition in both the tubulointerstitium and the glomerulus.

### Effect of triptolide on podocytes

Podocyte injury, characterized by foot process effacement (FPE), cytoskeletal disorganization, and loss of slit diaphragm proteins (nephrin and podocin), is the final common pathway to proteinuria in diverse glomerulopathies, including membranous nephropathy (MN), focal segmental glomerulosclerosis (FSGS), and DKD (Kopp et al. 2020). Agents such as puromycin aminonucleoside (PAN) and TGF-β are frequently used to induce podocyte damage. In a model of severe proteinuria induced by PAN injection in rats, PAN causes dose-dependent reorganization of the podocyte cytoskeleton, leading to excessive ROS production and upregulation of the injury marker desmin and. GADD45B is directly transactivated by p53, binds with NF-κB, and activates of MAPK, leading to podocyte injury (Chen et al. 2016; Yang et al. 2017). TP has emerged as a potent protector of podocytes, countering injury through a constellation of molecular mechanisms that stabilize the glomerular filtration barrier (Xu et al. 2015).

Oral administration of TP at 200 μg/kg significantly reduced PAN-induced proteinuria and improved FPE (Zheng et al. 2008). The efficacy of TP stems from its ability to simultaneously target oxidative stress, apoptotic signaling, and cytoskeletal integrity. A key mechanism involves the suppression of the p53/GADD45B axis. In a passive Heymann nephritis (PHN) model, TP (200 μg/kg, oral *in vivo*; 3 ng/mL *in vitro*) inhibits ROS generation, prevents cytoskeletal disruption, and restores the expression and localization of nephrin and podocin (Chen et al. 2010). In metronidazole -induced edema and proteinuria in zebrafish, pretreatment with 20 ng/ml TP blocked p53 binding to the GADD45B promoter, thereby suppressing GADD45B expression and its downstream activation of proapoptotic and inflammatory pathways (Wang et al. 2018a; Zhai et al. 2019). Similarly, TP counteracts TGF-β-induced damage by preventing Smad2/3 phosphorylation, nuclear translocation, and the downregulation of protective microRNA-30, thereby preserving cytoskeletal integrity (Yang et al. 2017). Furthermore, 1.80 ng/mL TP promotes genomic stability in podocytes by enhancing ten-eleven translocation 2 (TET2)-mediated DNA demethylation, which upregulates the expression of ZO-1, thereby reducing podocyte permeability (Tang et al. 2024). TP at 200 μg/kg protects against FSGS-related podocyte injury by promoting TET2-mediated demethylation of the NEPH1 and nephrin promoters, thereby enhancing slit diaphragm stability and integrity (Wan et al. 2021). Furthermore, TP inhibits NF-κB p65 activation and IκBα degradation in MN rats, reducing the expression of TNF-α, IL-1β, and CCL2 (Zhou et al. 2016). In PHN models, TP reduces 24-hour urine protein levels and alleviates renal and podocyte damage by inhibiting the PI3K/AKT pathway (Zhang et al. 2022). Another form of podocyte injury is induced by adriamycin (ADR), which commonly causes podocyte damage during clinical use (Liu et al. 2023). Studies have shown that angiopoietin-like 3 (Angptl3) is significantly upregulated in renal tissues of NS patients, and Angptl3 knockout mice exhibit renoprotective effects (Zhong et al. 2022). Pretreatment with 10 ng/ml TP was found to attenuate ADR-induced apoptosis in Angptl3 knockout primary podocytes (Ji et al. 2023).

TP demonstrates robust podocyte protection in diabetic models. In DKD mice, the width of podocyte foot processes is increased, accompanied by partial fusion of glomerular capillary loops and an increase in the thickness of the GBM. Treatment of DKD mice with TP (25 and 50 μg/kg) increased the podocyte density (Gao et al. 2010). An 8-week regimen with TP (100 μg/kg) significantly restored FPE and attenuated glomerular inflammation (Ma et al. 2013). In high-fat diet/streptozotocin-induced DKD, 50 μg/kg TP was shown to alleviate podocyte injury by inhibiting miR-155-5p, which upregulates brain-derived neurotrophic factor (BDNF) and suppresses inflammation/oxidative stress in HG-treated MPC5 cells (Gao et al. 2022). Intragastric TP (50 and 75 μg/kg) for 12 weeks alleviated renal structural injury and functional impairment by inhibiting podocyte EMT *via* the kindlin-2/TGF-β/Smad3 axis (Ren et al. 2022). TP (50 μg/kg *in vivo* and 1.80 ng/mL *in vitro*) activates the Nrf2 antioxidant pathway to inhibit ferroptosis, thereby preserving podocyte viability and structure (Wang et al. 2024).

In summary, TP confers broad-spectrum protection to podocytes through the targeting of multiple pivotal injury pathways. Its pleiotropic actions—spanning antioxidant, antiapoptotic, epigenetic, and anti-inflammatory activities—collectively preserve the cytoskeletal architecture, slit diaphragm integrity, and podocyte survival. This multitarget profile positions TP as a unique and promising therapeutic agent for podocytopathies that are often refractory to conventional single-pathway interventions.

### Effect of triptolide on mesangial cells

MCs are central to glomerular homeostasis, as they produce ECM and maintain capillary structure (Chaudhari et al. 2022; Deng et al. 2023). In diseases such as IgA nephropathy (IgAN) and DKD, pathological MCs activation—characterized by excessive proliferation, hypertrophy, and a transition toward a profibrotic myofibroblast phenotype—drives mesangial expansion, ECM overproduction, and a progressive decline in glomerular function (Boi et al. 2023).

#### Triptolide protects mesangial cells from abnormal proliferation

MCs proliferation is a key pathogenic driver in Ig AN, with mesangial alterations (from mild expansion to diffuse proliferation) closely linked to the clinical and pathological severity of the disease (Kim et al. 2024). TP counteracts this phenomenon via pleiotropic mechanisms. *In vitro*, TP (20 ng/mL) suppresses the expression of caspase recruitment domain family member 9 (CARD9), a regulator of autophagy and proliferation in human MCs (HMCs) (Zhao et al. 2022a). *In vivo*, intragastric administration of TP (300 μg/kg for 6 weeks) in murine IgAN models reduces glomerular IgA deposition, inhibits proliferation markers (PCNA and cyclin D1), and enhances autophagic flux (increased LC3II and decreased p62), thereby alleviating mesangial hyperplasia (Zhao et al. 2022a). Complementary studies in IgAN rat models have shown that 200 μg/kg TP also dampens MCs inflammation by reducing IL-1β and IL-18 levels and downregulating the toll-like receptor 4 (TLR4) and NACHT, LRR, and PYD domain-containing protein 3 (NLRP3) inflammasome pathway, contributing to reduced proteinuria and preserved renal function (He et al. 2015).

#### Triptolide protects mesangial cells from fibrosis

MCs proliferation in DKD frequently progresses to glomerular fibrosis, with the PI3K/Akt pathway being a primary therapeutic target of TP.

Firstly, 3-phosphoinositide-dependent protein kinase 1 (PDK1) phosphorylates Akt at the threonine 308 site, activating Akt and promoting cell proliferation (Mangé et al. 2019). TP suppresses HG-induced MCs proliferation by inhibiting the PDK1/Akt/PI3K axis, as reflected by reduced Ki-67 and PCNA expression (Han et al. 2017). TP upregulates the key phosphatase PTEN—a negative regulator of PI3K/Akt—by inhibiting the microRNAs that target it (miR-188-5p and miR-141-3p). This increase in the expression of PTEN promotes autophagy and decrease the expression of fibrotic markers (Col IV, FN, and α-SMA) (Li et al. 2017b; Xue et al. 2018). AKT, and PI3K converge on a common downstream target, leading to the inactivation of glycogen synthase kinase-3 beta (GSK-3β) (Zheng et al. 2020). Treatment with 50 μg/kg TP reduces GSK3β phosphorylation, activates the GSK-3β/nuclear factor erythroid 2-related factor 2 (Nrf2)/heme oxygenase-1 (HO-1) axis, alleviates renal dysfunction, and decreases fibrotic proteins (Col IV, TGF-β, and α-SMA) (Fan et al. 2023). TP has a synergistic effect with standard immunosuppressants. At 0.5 ng/ml, TP can potentiate the cytoskeleton-stabilizing effect of cyclosporine A, and preserving filtration barrier function, and alleviating diabetic injury *via* a GSK3β-dependent mechanism (Liang et al. 2020).

Additionally, 100 μg/kg TP upregulates the expression of miR-137, which directly targets and suppresses the expression of Notch1. This downregulation of Notch1 expression reduces the accumulation of ECM proteins (Col IV and FN), mitigating glomerulosclerosis (Han et al. 2018).

TP directly targets this central fibrogenic cascade. In TGF-β1-stimulated MCs, 10 ng/mL TP was shown to inhibit proliferation, downregulates Smad3, and upregulates the endogenous inhibitor Ski, altering their subcellular localization (Cao et al. 2015). This effect has been corroborated *in vivo*: 200 μg/kg TP attenuates fibrosis in a rat glomerulonephritis model. Similarly, in LPS-stimulated HMCs, TP inhibits proliferation, induces cell cycle arrest, and reduces the expression of α-SMA, TGF-β1, and phosphorylated Smad2/3, confirming its broad antifibrotic action *via* Smad pathway blockade (Cao et al. 2015).

In summary, TP exerts a concerted protective effect on MCs by targeting the dual pathological hallmarks of glomerular disease: aberrant proliferation and fibrosis. This phenomenon is achieved through a multipronged strategy involving the promotion of autophagic clearance, suppression of proliferative signaling (PI3K/Akt), inhibition of core fibrogenic pathways (TGF-β/Smad, Notch1), and activation of endogenous antioxidant defenses (GSK-3β/Nrf2/HO-1).

### Effect of triptolide on renal immune cells

The kidney is both a target and a regulator of the immune system. This intimate relationship makes it particularly vulnerable to immune-mediated injury, as exemplified by autoimmune conditions such as lupus nephritis (LN) and alloimmune responses following kidney transplantation (Li et al. 2023). TP exerts potent immunomodulatory effects that can mitigate such injury.

In LN, the deposition of immune complexes triggers complements activation and a robust inflammatory influx. Chemokines such as CXCL10 and CCL2 are crucial for recruiting proinflammatory cytokines (e.g., TNF-α and IL-6) and immune cells (T cells and macrophages) into the kidney (Puapatanakul et al. 2019; Gao et al. 2020). TP intervention disrupts this cascade. In a NZB/W F1 murine model of lupus, 15 μg/kg TP treatment reduced the production of TNF-α, IL-6, and CCL2, leading to improved renal outcomes and extended survival (Tao et al. 2008). Mechanistically, TP inhibits interferon-gamma (IFN-γ), thereby blocking the downstream JAK/STAT1 signaling pathway and suppressing the expression of the key chemokine CXCL10 in glomerular cells, which curtails leukocyte recruitment (Shi et al. 2023).

Immune dysregulation also contributes to the progression of DKD. TP exerts a dual immunoregulatory effect under these conditions. In DKD rats, a medium dose of TP (200 μg/kg) restored the imbalanced peripheral Th1/Th2 cell ratio, suppressed renal macrophage infiltration (marked by reduced CD68), and downregulated key proinflammatory (p-NF-κB, MCP-1) and profibrotic (TGF-β1) mediators. These immunomodulatory effects correlated with improved albuminuria and renal histology, confirming the role of TP in mitigating immune-driven injury in metabolic kidney disease (Guo et al. 2016).

For end-stage renal disease patients receiving kidney transplants, preventing rejection is paramount. Donor-specific antibodies (DSAs) are key mediators of antibody-mediated rejection (Aubert et al. 2017). TP, as an immunosuppressive agent, has been shown to reduce transplant rejection reactions. Research has indicated that its prodrug can alleviate DSA-mediated allograft injury by suppressing B cells, T cells, NK cells, and macrophages, thereby reducing inflammatory infiltration in the transplanted kidney and extending allograft survival (Zhao et al. 2018).

**Figure 2.**
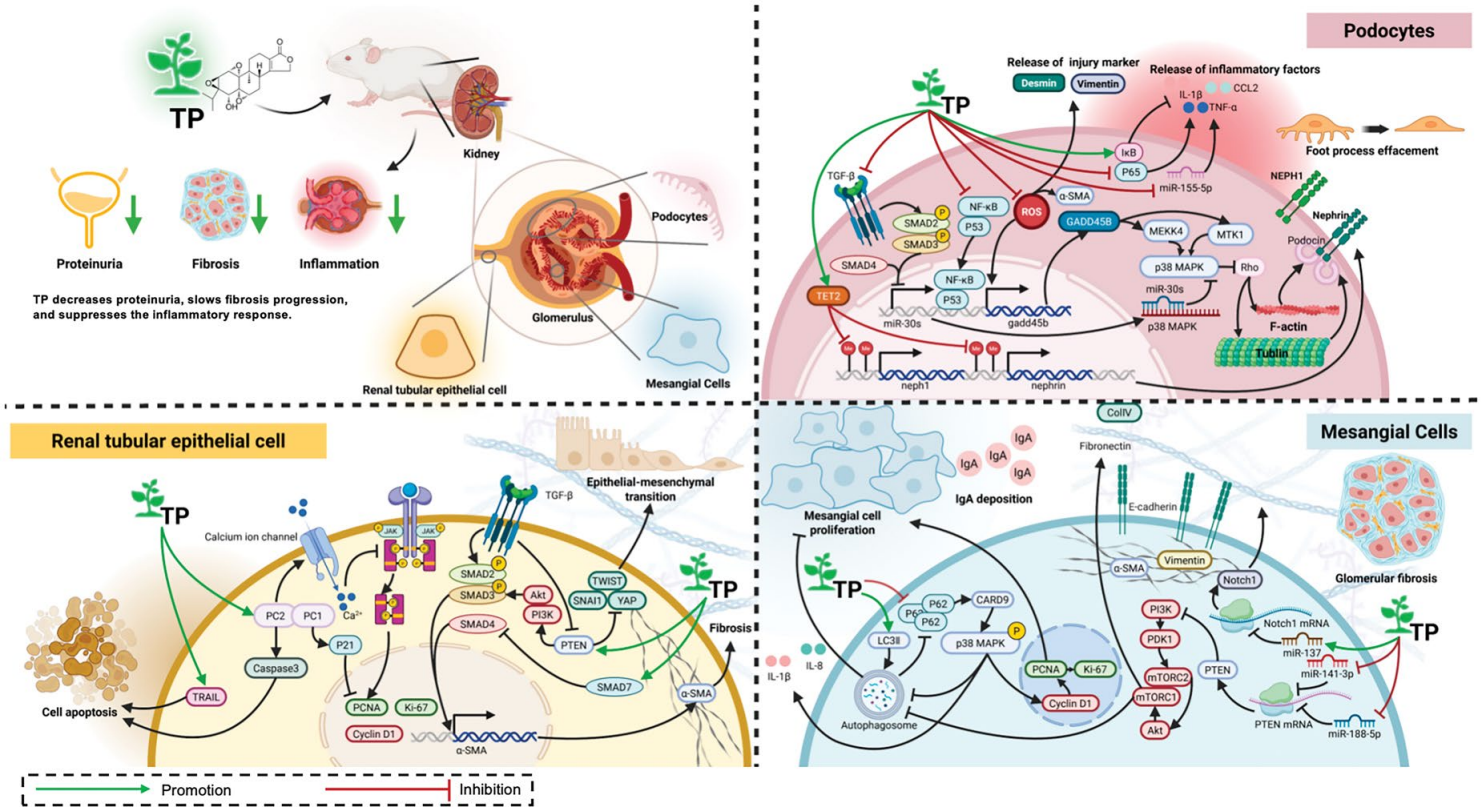
Triptolide in the treatment of kidney diseases. **Abb:** α-SMA, alpha-smooth muscle actin; AKT, Protein Kinase B; Bax, BCL2 Associated X; Bcl-2, B-cell lymphoma 2; CCL2, C-C motif chemokine ligand 2; Caspase, Cysteine-aspartic acid protease; CDK, Cyclin-dependent kinase; COX-2, Cyclooxygenase-2; ECM, Extracellular matrix; FcγRIIB, Fc gamma receptor IIB; F-actin, Filamentous actin; GADD45B, Growth arrest and DNA damage-inducible beta; IgA, Immunoglobulin A; IL-1β, Interleukin-1 beta; JAK/STAT, Janus kinase/Signal transducer and activator of transcription; LC3B, Microtubule-associated protein 1 light chain 3 beta; MAPK, Mitogen-activated protein kinase; miR, MicroRNA; mTOR, Mammalian target of rapamycin; NF-κB, Nuclear factor kappa-B; NR4A1, Nuclear receptor subfamily 4 group A member 1; PCNA, Proliferating cell nuclear antigen; PDK1, 3-Phosphoinositide-dependent protein kinase 1; PI3K, Phosphatidylinositol 3-kinase; PTEN, Phosphatase and tensin homolog deleted on chromosome ten; ROS, Reactive oxygen species; TGF-β, Transforming growth factor-beta; TLR, Toll-like receptor; TP, Triptolide; TRAIL, Tumor necrosis factor-related apoptosis-inducing ligand; Vimentin, Vimentin; β-catenin, Beta-catenin

TP has distinct therapeutic effects across major kidney cell types: in renal tubular epithelial cells, it suppresses abnormal proliferation and inhibits fibrosis; in podocytes, it stabilizes the filtration barrier and reduces proteinuria; and in mesangial cells, it curbs pathological expansion and extracellular matrix production. These cell-specific actions, combined with its broad immunomodulatory capacity, underscore the potential of TP as a multitarget agent for diverse kidney disorders, although its clinical translation is constrained by a narrow therapeutic window (Figure 2, Table 1).

**Table 1.** The therapeutic benefits and pharmacological mechanisms of triptolide in renal diseases models.

Diseases	Model	TP dosage	Duration	Administration	Indication	Mechanisms	Ref.
PKD	Pkd2-/-Murine kidney epithelial cells	36.04 ng/ml	96 h	NR	Cell number↓	Promote apoptosis	(Leuenroth et al. 2007)
	*Pkd1^flox/-^; Ksp-Cre* model	120 μg/kg 250 μg/kg	4 days	ip	number of cysts↓, kidney weight of cystic animals↓ Ki-67↓	Promote apoptosis	(Leuenroth et al. 2008)
	*Pkd1^flox/flox^*; Mx1Cre model	120 μg/kg 250 μg/kg	20 days	iv	Frequency distribution of the number of cysts ↓, BUN↓,	Inhibit cyst early proliferative	(Leuenroth et al. 2010)
	Han:SPRD rats	200 μg/kg	12 weeks	ig	BUN↓, Scr↓, urine proteins↓	Promote apoptosis	(Jing et al. 2018)
	WT 9–12 cells	108.12 ng/ml	48 h	NR	p-JAK2/JAK2↓, p-STAT3/STAT3↓, apoptotic cell↑		
RCC	786–0 OS–RC–2	18.02 ng/ml	24 h	NR	caspase-3↑, Cyto c, Bax, Bcl-2↓, Bcl-Xl↓	Promote apoptosis	(Li et al. 2011)
	ACHN A498 Caki-1 769-P 786-O	18.02 ng/ml, 36.04 ng/ml	24 h	NR	TRAIL-R1↑, TRAIL-R2↑, TRAIL-R3↑, TRAIL-R4↑, HSP70↓,	Promote apoptosis	(Brincks et al. 2015)
DKD	HKC cells exposed to IFN-γ and TNF-α	10 ng/ml	24 h	NR	class II MHC↓, B7-1↓, B7-2↓	Inhibit inflammation	(Li et al. 2002).
	NRK-49F cultured with TGF-β1	10 ng/ml	6-12 h	NR	Smad2↓	Inhibit ECM synthesis	(Zhu et al. 2010)
	Human PTEC clone cultured with TNF-α	2-8 ng/ml	24 h	NR	B7-1↓	Inhibit inflammation	(Hong et al. 2002)
	DKD mice	25 μg/kg 50 μg/kg	4 weeks, 8 weeks 12 weeks	NR	urine albumin↓, desmin↓,	Inhibit inflammation	(Gao et al. 2010)
	DKD rats	50 μg/kg	8 weeks	ig	FBG↓, BUN↓, Scr↓, UACR↓	Inhibit OS and inhibit renal fibrosis	(Fan et al. 2023)
	SV40-MES-13 cells cultured with glucose	2 ng/mL	48 h	NR	Col IV↓, TGF-β↓, α-SMA↓, NOX4↓, SOD↑, p-GSK3β↓, Nrf2↑, HO-1↑,		
	DKD mice	50 μg/kg	8 weeks	ip	GPX4↑, FTH-1↑, SLC7A11↑, TFR-1↓, Nrf2↑	Inhibit ferroptosis	(Wang et al. 2024).
	HPCs cultured with glucose	1.80 ng/mL	48 h	NR			
	DKD mice	50 μg/kg 75 μg/kg	12weeks	ig	nephrin↑, podocin↑, E-cadherin↑, α-SMA↓, TGF-β1, p-SMAD3, kindlin-2	Inhibit renal fibrosis	(Ren et al. 2022)
	DKD mice	50 μg/kg	12weeks	ig	24 h UMA↓, miR-155↓, Nephrin↑	Inhibit inflammation and OS	(Gao et al. 2022)
	MPC5 cultured with glucose	3604 ng/ml	48 h	NR	miR-155↓		
	DKD rats	100 μg/kg	8 weeks	ig	albuminuria↓	Inhibit inflammation	(Ma et al. 2013)
	DKD rats	100 μg/kg	12 weeks	ig	KW/BW↓	Inhibit mesangial cell proliferation	(Han et al. 2017)
	DKD rats	100 μg/kg	12 weeks	ig	Albuminuria↓, Col IV↓, FN↓, Notch1↓, miR-137 ↑	Inhibit mesangial cell proliferation	(Han et al. 2018)
	HRMC cultured with glucose	10 ng/mL	12 h, 24 h 48 h 72 h	NR	Cell viability↓, G0/G1↑, G2/M↓, p-AKT/AKT↓, p-mTOR/mTOR↓, Ki-67↓, PCNA↓ PDK1↓		
DKD	DKD mice	100 μg/kg	6 weeks	ig	Urine↓, BUN↓, ACR↓	Inhibit renal fibrosis	(Li et al. 2019)
	DKD rats	200 μg/kg	12 weeks	ig	E-cadherin↑, Vimentin↓, α-SMA↓, PTEN↑, PI3K↓, p-AKT↓, miR-188-5p↓	Inhibit renal fibrosis	(Xue et al. 2018)
	HK-2 cultured with glucose	5 ng/mL	48 h	NR			
	DKD rats	200 μg/kg	4 weeks	ig	24 h urine protein↓, Urine nitrogen↓, Creatinine↓	Inhibit mesangial cell proliferation	(Cao et al. 2015)
	HBZY-1 cultured with TGF-β1	10 ng/ml	48 h	NR	Smad3↓, Ski↑		
	DKD rats	200 μg/kg	12 weeks	ig	Col IV↓, FN↓, p62↓, LC3-II/LC3-I↑, Atg 5↑, miR-141-3p↓, PTEN↑, p-AKT/AKT↓, p-mTOR/mTOR↓	Promote autophagy and inhibit renal fibrosis	(Li et al. 2017b)
	HRMC cultured with glucose	3604 ng/ml	48 h	NR			
	DKD rats	200 μg/kg 300 μg/kg 400 μg/kg	4 weeks	ig	FBG↓, BUN↓, Scr↓	Inhibit renal fibrosis	(Pang and Gu 2021)
	UUO rats	600 μg/kg	1 weeks	ig	TGF-β1↓, CTGF↓, MCP-1↓, osteopontin↓	Inhibit renal fibrosis	(Yuan et al. 2011)
	DKD rats	6000 μg/kg 12000 μg/kg 24000 μg/kg	4 weeks	ig	KW/BW↓, IFN-γ↓, TNF-α↓, IL-4↓, IL-10↓	Regulate Th Cell Balance and Macrophage Infiltration	(Guo et al. 2016).
MN	HPCs stimulated by PAN	1.80 ng/mL	NR	NR	ZO-1↑, TET2↑	Regulating TET2-mediated hydroxymethylation	(Tang et al. 2024)
	cBSA rat models	200 μg/kg	4 weeks	iv	24hUTP↓, TNF-α↓, IL-1β↓, MCP-1↓, MDA↓, SOD↑	Inhibit inflammation and OS	(Zhou et al. 2016)
	PAN-induced podocytes injury	200 μg/kg	3 weeks	ig	proteinuria↓	Inhibit OS	(Zheng et al. 2008)
	Podocytes stimulated by PAN	3 ng/ml	30 min	NR	nephrin↑, podocin↑		
	PHN rat models	200 μg/kg	4 weeks	ig	urinary protein↓, IgG deposition in glomeruli↓, C5b-9 deposition↓, Nephrin↑	Inhibit OS	(Chen et al. 2010)
	Mouse podocytes stimulated by IFN-γ	10 ng/ml	30 min	NR	ROS↓		
FSGS	FSGS rats	200 μg/kg	8 weeks	ig	nephrin↑, NEPH1↑, α-SMA↓, TET2↑	Regulating TET2-mediated hydroxymethylation	(Wan et al. 2021).
IgAN	TMC from IgAN patients	10 ng/mL 20 ng/mL 30 ng/mL	24 h	NR	Cell viability↓, apoptotic cells ↑, BCL-2↓, Bax↑	Promote apoptosis	(Yan et al. 2016)
	IgAN mice	200 μg/kg	14 weeks	ig	Proteinuria↓, Urine protein:creatinine ratio↓, IgA deposition↓,		(He et al. 2015)
	IgAN mice	300 μg/kg	6 weeks	ig	IgA in glomeruli↓, BUN↓, PCR↓, SCr↓, UA↓	Promotes autophagy and inhibit mesangial cell proliferation	(Zhao et al. 2022a)
	HRMC incubated with IgA1	20 ng/mL	24 h	NR	LC 3II↑, PCNA↓, cyclin 1↓, CARD9↓, LC3II/LC3I↑, PCNA ↓		
LN	NZB X NZW F1/J mic	15 μg/kg	15 weeks	ig	Proteinuria↓, BUN↓, Scr↓, IgG↓, anti-dsDNA↓, C3↓, IL-6↓, MCP-1↓	Inhibit inflammation	(Tao et al. 2008)
	MMC	5 ng/ml 10 ng/ml 20 ng/ml	48 h	NR	CXCL10↓	Inhibit inflammation	(Shi et al. 2023)
AKI	CD19^+^ B cells purified from human peripheral mononuclear leukocytes	4 ng/mL 40 ng/mL	2-8 days	NR	B cell apoptosis↑	Promote apoptosis	(Zhao et al. 2018)
	Angptl3 knockout primary podocytes	10 ng/ml	30 min	NR	podocin↑, cd2ap ↑, apoptosis rate ↓	Promote apoptosis	(Ji et al. 2023)

**Abb:** 24hUTP, 24-hour urine total protein quantity; AKI, Acute kidney injury; Angptl3, Angiopoietin-like 3; BUN, blood urea nitrogen; cBSA, cationic bovine serum albumin; CARD9, Caspase recruitment domain-containing protein 9; CXCL10, C-X-C motif chemokine 10; DKD, diabetic kidney diseases; GPX4, Glutathione peroxidase 4; GSK-3β, Glycogen synthase kinase-3 beta; HO-1, Haem oxygenase-1; HRMC, Human renal mesangial cells; ig, Intragastric administration; IL-1β, Interleukin-1β; ip, intraperitoneal administration; iv, intravenous administration; KW/BW, Kidney weight to body weight ratio; LN, lupus nephritis; MMC, Mouse glomerular mesangial cells; MN, membranous nephropathy; NLRP3, NACHT, LRR, and PYD domains-containing protein 3; NOX4, NADPH oxidase 4; NR, not report; Nrf2, Nuclear factor erythroid 2-related factor 2; OS, Oxidative stress; PCNA, Proliferating cell nuclear antigen; PCR, ratios of urine protein/creatinine; PHN, Passive Heymann nephritis; PKD, Polycystic kidney disease; p-JAK2, phosphorylated JAK2; p-STAT1, phosphorylation-STAT; p-STAT3, phosphorylated STAT3; RCC, Renal cell carcinoma; SCr, serum creatinine; SLC7A11, Solute carrier family 7 member 11; SOD, Superoxide dismutase; TGF-β, Transforming growth factor-β; TLR4, Toll-like receptor 4; TMC, tonsillar mononuclear cells; TP, Triptolide; TRAIL, TNF-associated apoptosis-inducing ligand; UA, uric acid; UMA, urinary microalbumin.

## Nephrotoxicity of triptolide on renal tubular epithelial cells

Despite its therapeutic potential in kidney diseases, TP is nephrotoxic at supratherapeutic doses or with prolonged administration, posing a critical challenge to its application. Numerous reports have documented severe renal damage caused by TP overdose or improper administration, which has drawn significant attention from researchers (Yang et al. 2018). Long-term use of TP in some patients has been associated with adverse effects such as fatal acute renal failure. Data from the China National Adverse Drug Reaction Monitoring Center indicate that 839 cases of adverse reactions to TwHF were reported between 2004 and 2011, with 9% classified as severe and involving significant renal impairment (Li et al. 2021). Among the bioactive components of TwHF, TP has been identified as the primary contributor to its nephrotoxicity through comparative toxicity studies (Li et al. 2015), making it critical to investigate the mechanisms underlying TP-induced renal injury. TP derivatives identified a relevant Phase II clinical trial of Minnelide (Min), a water-soluble prodrug of triptolide, which in patients with advanced refractory biliary tract cancer. In this trial, 12 patients were enrolled, among whom 16.67% (2/12) experienced elevated serum creatinine (Scr) levels as a treatment-emergent adverse event, indicating potential renal function alteration associated with the derivative (Skorupan et al. 2022). Experimental evidence suggests that TP selectively targets RTECs, thereby disrupting renal function.

### TP alters the structure and function of renal tubular epithelial cells

Nephrotoxic drugs enter RTECs through two primary pathways: endocytosis and pinocytosis, or active transport *via* organic anion/cation transporters located at the basolateral membrane of proximal tubule epithelial cells (PTECs) (Perazella 2019). This dual-entry mechanism leads to intracellular drug accumulation, ultimately impairing renal tubular function. Key markers involved in these processes include sodium-glucose cotransporter 2 (SGLT2), organic cation transporter 2 (OCT2), zonula occludens 1 (ZO-1), and junctional adhesion molecule 1 (JAM-1), which are critical for drug excretion by RTECs (Petreski et al. 2025).

OCT2 plays a key role in this process. In a collagen-induced arthritis model, TP at 500 μg/kg exacerbates renal accumulation by upregulating OCT2 expression. This process increases renal TNF-α levels, despite the ability of TP to alleviate joint swelling (Shen et al. 2020). Single-cell RNA sequencing revealed that C57BL/6 mice treated with TP at 500 μg/kg *via* intraperitoneal injection for two weeks exhibited significant downregulation of tight junction proteins in proximal tubules, impairing barrier integrity and tubular function (Wu et al. 2024). *In vivo*, oral administration of TP at 100, 200, and 400 μg/kg in Wistar rats dose-dependently elevated blood urea nitrogen (BUN) and Scr levels, indicating renal dysfunction (Sun et al. 2013; Luo et al. 2023). Functionally, *in vitro*, 30 nM TP downregulated SGLT1 and SGLT2 expression in HK-2 cells, reducing glucose uptake (Wang et al. 2009).

### TP promotes inflammation and oxidative stress of renal tubular epithelial cells

TP-induced nephrotoxicity is closely linked to the disruption of the Nrf2-KEAP1 system, a central regulator of cellular antioxidant defenses. Impairment of Nrf2 function not only exacerbates oxidative stress but also relieves its tonic inhibition of the proinflammatory cyclic GMP-AMP synthase/stimulator of interferon genes (cGAS/STING) pathway (Liu et al. 2022). This dual regulation positions Nrf2 as a key node balancing oxidative stress and inflammation in renal homeostasis.

TP rapidly induces a cascade of oxidative and inflammatory events that are central to its nephrotoxicity. A single intraperitoneal injection of TP (500 μg/kg) in mice was shown to significantly elevate renal ROS levels within a remarkably short timeframe of 15 min (Liu et al. 2021). Sustained exposure (500 μg/kg *via* intraperitoneal injection for 6 days-2 weeks) disrupted the NRF2-BACH1 balance, impairing antioxidant defenses and activating the cGAS-STING pathway, leading to a type I interferon response (Lu et al. 2022; Wu et al. 2024). Furthermore, a recent study in SD rats receiving TP *via* gavage linked this oxidative-inflammatory axis to impaired mitophagy, identifying TBK1 downregulation as a key event. Critically, TBK1 overexpression *in vitro* and *in vivo* reversed TP-induced mitophagy suppression and tubular injury, confirming its pivotal role in the nephrotoxic mechanism. The synergistic activation of oxidative stress and inflammatory pathways ultimately induces RTECs apoptosis and propagates systemic inflammatory responses, exacerbating TP-induced renal damage (Wang et al. 2019). Pretreatment with the antioxidant vitamin C significantly mitigated TP-induced oxidative stress and renal functional impairment, confirming the central role of oxidative stress in TP nephrotoxicity (Yang et al. 2011, 2012), while pretreatment with a GSH inhibitor exacerbates TP-induced renal toxicity (Li et al. 2015), confirming the central role of oxidative stress in TP nephrotoxicity.

### TP affects metabolic dysregulation

TP nephrotoxicity is also linked to systemic metabolic disturbances. In a delayed-type hypersensitivity model, a therapeutic dose of TP (450 μg/kg via intraperitoneal injection) predominantly generated phase I metabolites, whereas a toxic dose (900 μg/kg *via* intraperitoneal injection) led to glutathione depletion and a shift toward phase II conjugates, indicating an overwhelmed detoxification capacity (Wang et al. 2018b)^.^

Metabolomic studies in Wistar rats showed that both subacute exposure (1000 μg/kg by gavage for 10 days) and longer-term exposure (200 μg/kg for 28 days) induce purine metabolism abnormalities, characterized by decreased serum adenosine and elevated adenosine deaminase activity, which in turn activated the TLR-NF-κB pathway and promoted inflammation (Huang et al. 2019b; Xie et al. 2020). Additionally, TP disrupted fatty acid metabolism-related protein expression, impairing renal energy homeostasis (Li et al. 2017a).

**Figure 3.**
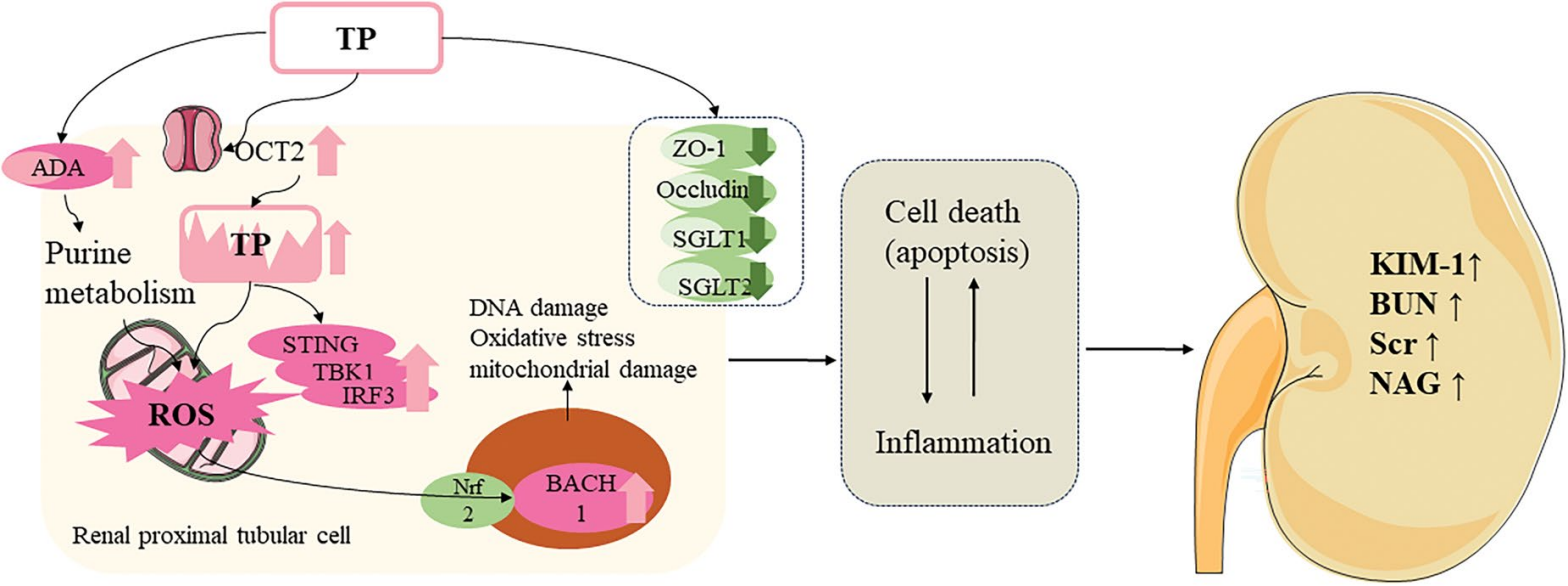
Mechanism of nephrotoxicity induced by triptolide. **Abb:** BACH1, BTB and CNC homology 1; BUN, Blood urea nitrogen; IRF3, Interferon regulatory factor 3; KIM-1, Kidney injury molecule 1; NAG, N-­acetyl-β-D-glucosaminidase; Nrf2, Nuclear factor erythroid 2-related factor 2; OCT2, Organic cation transporter 2; ROS, Reactive oxygen species; Scr, Serum creatinine; STING, Stimulator of interferon genes; TBK1, TANK-binding kinase 1; TP, Triptolide; ZO-1, Zonula Occludens 1.

In summary, TP-induced nephrotoxicity involves a multifaceted cascade of structural and functional tubular damage, oxidative stress-inflammatory activation, and systemic metabolic dysregulation. These findings underscore the importance of precise dose control, renal function monitoring, and the development of targeted delivery strategies to enhance the safety profile of TP in clinical applications (Figure 3, Table 2).

**Table 2.** Summary of *in vivo* and *in vitro* studies on triptolide-induced nephrotoxicity.

Experimental subject	Model	TP dosage	Duration	Administration	Nephrotoxicity mechanisms	Results	Ref
*In vivo*	SD rats	200 μg/kg 400 μg/kg	28 days	ig	TP disrupts the structure and function of renal cells	Scr↑, renal tubules atrophy, Per1↓	(Luo et al. 2023)
	Wistar rats	200 μg/kg 400 μg/kg	28 days	ig	TP disrupts the structure and function of renal cells	ZO-1↓, Scr↑, BUN↑	(Sun et al. 2013)
	Wistar rats	200 μg/kg 400 μg/kg	28 days	ig	Affect purine metabolism disorders	Scr↑, BUN↑, ADA↑	(Xie et al. 2020)
	BALB/c mice	200 μg/kg 400 μg/kg	6 days	ip	Promote Oxidative stress	STING↑, TBK1↑, IRF3↑, IFNβ↑	(Lu et al. 2022)
	BALB/c mice	500 μg/kg 1000 μg/kg	15 min 30 min 1 h 48 h	ip	Promote oxidative stress and apoptosis	Scr↑, BUN↑, ROS↑, BCL-2↓, Bax ↑	(Liu et al. 2021)
	C57BL/6 mice	500 μg/kg	2 weeks	ip	Promote oxidative stress and apoptosis	Cxcl1↑, Cxcl10↑	(Wu et al. 2024).
	CIA rats	500 μg/kg	4 weeks	ig	TP accumulate in rat kidney	Scr↑, Kim-1↑, kidney TP↑, OCT2↑	(Shen et al. 2020)
	DTH Balb\c mouse	900 μg/kg	20 h	ig	Regulate SPL metabolism	Cer-1-p↓, Cer↑, HexCer↓	(Qu et al. 2015)
	BALB/c mice	1000 μg/kg	24 h	ip	Promote oxidative stress and apoptosis	ROS↑, LDH↑, Scr↑, BUN↑, MDA↑, SOD↓, GSH↓, Nrf2↑, NQO1↑, HO-1↑	(Wang et al. 2019)
	Wistar rats	1000 μg/kg	10 days	ig	Promote oxidative stress	Uric acid ↓, Xanthine↓, ROS↑	(Huang et al. 2019b)
	SD rats	1000 μg/kg	1 day	ip	Promote Oxidative stress	KW/BW↓, Apoptotic index↑, Cre↑, BUN↑, ATN score↑, SOD↓, GSH-Px↓, MDA↑	(Yang et al. 2012)
	SD rats	1000 μg/kg	1 day	ip	Promote Oxidative stress	KW/BW↓, ROS↑	(Yang et al. 2011)
	KM mice	1200 μg/kg	1 day	ig	Promote Oxidative stress	Mortality↑	(Li et al. 2015)
	C57BL/6J mice	1000 μg/kg 2000 μg/kg	24 h	ip	Promote inflammation	Scr↑, BUN↑, LDH↑, IL-6↑, MCP-1↑	(Zhang et al. 2023)
*In vitro*	HK-2 cells	18 ng/ml	24 h	NR	Promote oxidative stress and apoptosis	Cell viability ↓, the apoptotic cell ↑, LDH↑ MDA↑, SOD↓, GSH↓, Nrf2↑, NQO1↑, HO-1↑,	(Wang et al. 2019)
	HK-2 and HEK-293T stimulated by TNF-α or IL-1β	3.6 ng/ml	10 min	NR	TP accumulate in rat kidney	Uptake of TP↑, OCT2↑	(Shen et al. 2020)
	NRK-52E cells	3.6 ng/mL 9.0 ng/mL 18.0 ng/mL	24 h	NR	TP disrupts the structure and function of renal cells	JAM-1↓, ZO-1↓, and Occludin↓	(Sun et al. 2013)
	HK-2 and HKC cell	3.6 ng/mL 10.8 ng/mL	24 h 48 h 72 h	NR	Promote OS and disrupts the function of renal cells	LDH↑, NAG↑, Glucose uptake↓, SGLT1↓, and SGLT2↓	(Wang et al. 2009)
	HK-2 and HKC cell	7.2 ng/mL	48 h	NR	Promote Oxidative stress	p-STING↑, p-TBK1↑, IRF3↑, BACH1↑	(Lu et al. 2022)

**Abb:** ADA, Adenosine Deaminase; ATN, Acute Tubular Necrosis; BACH1, BTB and CNC homology 1; Bax, Bcl-2-associated X protein; BCL-2, B-cell lymphoma 2; BUN, Blood Urea Nitrogen; Cer, Ceramide; Cer-1-p, Ceramide-1-phosphate; CIA, Collagen-induced Arthritis; Cxcl1, C-X-C motif chemokine ligand 1; Cxcl10, C-X-C motif chemokine ligand 10; DTH, Delayed-type Hypersensitivity; GSH, Glutathione; GSH-Px, Glutathione Peroxidase; HexCer, Hexosylceramide; HO-1, Heme Oxygenase-1; IFNβ, Interferon-beta; ig, Intragastric administration; IL-1β, Interleukin-1β; IL-6, Interleukin-6; ip, Intraperitoneal administration; IRF3, Interferon Regulatory Factor 3; JAM-1, Junctional Adhesion Molecule 1; Kim-1, Kidney Injury Molecule-1; KW/BW, Kidney Weight to Body Weight ratio; LDH, Lactate Dehydrogenase; MCP-1, Monocyte Chemoattractant Protein-1; MDA, Malondialdehyde; NAG, N-Acetyl-β-D-glucosaminidase; NR, Not Reported; NQO1, NAD(P)H Quinone Dehydrogenase 1; Nrf2, Nuclear factor erythroid 2-related factor 2; OCT2, Organic Cation Transporter 2; OS, Oxidative Stress; Occludin, Occludin; p-STING, Phosphorylated STING; p-TBK1, Phosphorylated TANK-binding kinase 1; Per1, Period Circadian Regulator 1; ROS, Reactive Oxygen Species; Scr, Serum Creatinine; SGLT1, Sodium/Glucose Cotransporter 1; SGLT2, Sodium/Glucose Cotransporter 2; SOD, Superoxide Dismutase; SPL, Sphingolipid; STING, Stimulator of Interferon Genes; TBK1, TANK-binding kinase 1; TNF-α, Tumor Necrosis Factor-alpha; TP, Triptolide; ZO-1, Zonula Occludens-1.

## Methods for treating Triptolide in the kidneys

### Drug delivery system

Researchers have explored drug delivery systems with diverse carriers to enhance TP efficacy and reduce toxicity, with a focus on RTECs-targeted strategies.

Nanoparticle-based systems improve pharmacokinetics and renal accumulation. For instance, kidney-targeted mesoscale nanoparticles encapsulating TP (TP-MNPs) have been developed for the treatment renal ischemia-reperfusion injury (IRI). By specifically accumulating in RTECs, TP-MNPs increase the intracellular drug concentration while minimizing systemic exposure. Compared to free TP (500 μg/kg), TP-MNPs (50 μg/kg) demonstrate superior protection against IRI and significantly reduce neutrophil gelatinase-associated lipocalin (NGAL) (Deng et al. 2019). Compared to free TP, TP-loaded solid lipid nanoparticles (TP-SLN) increase the area under the curve (AUC) and mean residence time in rats. This sustained-release profile reduces peak plasma concentrations while maintaining effective renal exposure (Xue et al. 2012), and a poly-γ-glutamic acid-based nanosystem (γ-PGA-l-PAE-TP, PPT) facilitates hepatic metabolism of free TP and accelerates renal excretion of metabolites. By reducing the accumulation of unmetabolized TP in renal tissues, PPT decreases TP-induced apoptosis in multiple renal cell types (including RTECs) and decreases the required dosage and administration frequency (Zhang et al. 2016). Folate-modified nanoparticles (TP-FPNPs) leverage folate receptor-mediated endocytosis to enhance renal targeting and reduce systemic toxicity (Huang et al. 2019a).

Ligand-receptor-targeted conjugates employ molecular recognition for precise delivery. TP as the receptor and 2-glucosamine as the glycosyl donor (targeting ligand) to synthesize triptolide aminoglycoside (TPAG), enabling active targeting of TP to the kidneys. Compared with free TP, TPAG shows higher renal AUC, providing enhanced protection against IRI (Qi et al. 2015). Similarly, compared with free TP, the triptolide-glucosamine conjugate (TPG) achieves superior renal targeting through megalin receptor interactions, with *in vitro* studies showing that HK-2 cells take up TPG more efficiently and avoid TP-induced cell cycle arrest and apoptosis. In this study, triptolide-glucosamine conjugate (TPG) was synthesized by linking TP to 2-glucosamine *via* a carbamate bond; this conjugate exhibited superior renal targeting efficiency, with a renal AUC greater than that of free TP in mice. Notably, by maintaining normal BUN levels, minimizing tubular degeneration/necrosis, and reducing the renal apoptotic index, TPG not only showed enhanced protective effects against IRI in rats but also offers a promising approach to balance its therapeutic efficacy and safety in renal disorders (Zhou et al. 2014; Fu et al. 2016). A particularly promising approach exploits the inherent renal tropism of lysozyme (LZM). Lysozyme-drug conjugates, such as 14-succinyl triptolide-lysozyme (TPS-LZM), are filtered by the glomerulus and selectively reabsorbed by PTECs *via* receptor-mediated endocytosis. Notably, TPS-LZM is efficiently taken up by HK-2 cells, increasing intracellular TP concentrations and enhancing efficacy in IRI models. TPS-LZM at an equivalent TP dose of 10 μg/kg achieves the reductions in Scr and NGAL, while free TP requires 500 μg/kg to reach comparable efficacy (Zhang et al. 2009; Deng et al. 2019). Recent advancements have further optimized this strategy by formulating LZM-functionalized cationic liposomes (LZM-PLNPs-TP). This design bypasses the glomerular filtration barrier and targets tubules *via* peritubular capillaries. In a mouse IRI model, LZM-PLNPs-TP (equipped TP 10 μg/kg) significantly enhances renal accumulation and outperforms free TP at the same dose in mitigating oxidative stress, inflammation, and functional impairment (Guo et al. 2025).

Advanced targeting strategies address specific clinical challenges. In childhood nephrotic syndrome, a γ-glutamyltransferase-responsive dendrimer-drug conjugate (GSHPD) exploits elevated glomerular γ-glutamyltransferase (GGT) expression for enzyme-triggered TP release, showing significant renal function recovery in juvenile rat models (Chen et al. 2024). For CKD, biomimetic high-density lipoprotein nanoparticles (bHDLs) coloaded with TP and nintedanib target injured tubular cells *via* KIM-1 recognition, demonstrating synergistic antifibrotic effects with reduced TP toxicity (He et al. 2025). Peptide-mediated delivery using human serum albumin fragments (TPS-PF-A299-585) shows effective renal enrichment and maintenance of anti-inflammatory activity with lower cytotoxicity (Yuan et al. 2015). With respect to mesangial-specific delivery in glomerular diseases, TRX-20-modified liposomes (PEG-TRX-TP-LP) effectively target MCs and improve outcomes in membranous nephropathy models (Yuan et al. 2017).

### Structural modification

While direct human data on purified TP are lacking, the consistent signal from herbal pharmacovigilance data, reinforced by robust experimental evidence pinpointing TP as the key toxic agent, provides a compelling case for its nephrotoxic potential. Furthermore, clinical trials of its derivatives offer a bridge, confirming that the toxic mechanisms identified in preclinical studies are relevant to human patients. Owing to the poor water solubility, low oral bioavailability, and multiorgan toxicity of TP, researchers have recently designed various TP derivatives through structural modifications. These derivatives have shown varying degrees of progress in reducing toxicity and enhancing efficacy in the treatment of kidney diseases. Among them, LLDT-8, PG490-88, and Min are the most promising (Zeng et al. 2023).

PG490-88 is generated *via* carboxylation of the C-14 hydroxyl group, a modification that enhances water solubility and reduces nonspecific cytotoxicity. This derivative can protect PTECs by inhibiting the MAPK/ERK pathway, which is hyperactivated in cisplatin-induced AKI (Kim et al. 2014).

LLDT-8 is synthesized by introducing a hydroxyl group at the C-5 position, replacing a hydrogen bond to modulate interactions with immune receptors. This modification redirects the activity of TP toward glomerular cells, including podocytes and MCs, by enhancing its anti-inflammatory effects (Zhou et al. 2005). In anti-GBM nephritis models, LLDT-8 restores the expression of the inhibitory Fc receptor Fc gamma receptor IIB (FcγRIIB) on glomerular immune cells, counteracting the upregulation of the activator Fc receptor Fc FcγRI/III (Qi et al. 2018). This phenomenon shifts the FcγR balance to inhibit ICs deposition, complement activation, and proinflammatory cytokine (TNF-α, IL-6) release, thereby reducing proteinuria and preserving GFB integrity (Qi et al. 2018). Similarly, in MRL/lpr mice (a model of LN), LLDT-8 decreases glomerular IgG deposition and suppresses the expression of chemokines (CXCL9/10), limiting T-cell/macrophage infiltration into glomeruli (Zhang et al. 2017).

Min incorporates a phosphate group to enhance water solubility, enabling broader distribution across renal cell types. It protects glomerular cells and restores the foot processes in mice with ADR-induced NS. Min normalized the localization of key slit diaphragm proteins such as nephrin, podocin and cd2ap and mitigates the expression of inflammatory factors (TNF-α, IL-6 and IL-1β) (Ji et al. 2023). Notably, it exerted its renoprotective effects by suppressing the TGF-β1-Smad2 and p53 signaling cascades, which drive fibrosis and apoptosis in diverse kidney diseases (Ji et al. 2023). Min was examined in a mouse model of ADR-induced nephropathy and in PAN-damaged primary podocytes. Intraperitoneal administration of Min for 2 weeks significantly reduced proteinuria and renal apoptosis in ADR-nephropathy mice, while *in vitro*, TP alleviated PAN-induced podocyte cytoskeletal rearrangement and apoptosis *via* the ROS-mediated mitochondrial pathway with no reproductive toxicity in male and female mice.

### Combination therapy

Combination therapy offers a promising strategy to address narrow therapeutic window of TP by either synergistically enhancing its renal protective efficacy or antagonistically mitigating nephrotoxicity (Das et al. 2019). This approach is particularly valuable for TP, as its therapeutic doses are close to toxic thresholds, and targeted combinations can uncouple efficacy from toxicity (Cao et al. 2022).

A well-characterized example is the coadministration of TP with *Panax notoginseng (Burkill) F.H.Chen (Araliaceae)*, commonly known as Sanqi, and *Rehmannia glutinosa (Gaertn.) DC. (Orobanchaceae)*, known as Dihuang, two traditional Chinese medicines with nephroprotective properties. In rat models, this combination reduces TP-induced elevations in BUN and Scr levels and alleviates renal tubular damage by extending the half-life of TP (from 7.07 to 9.31 h) and increase its apparent distribution volume, thereby lowering peak plasma concentrations while maintaining effective renal exposure (Zhang et al. 2018).

## Discussion and summary

**Figure 4.**
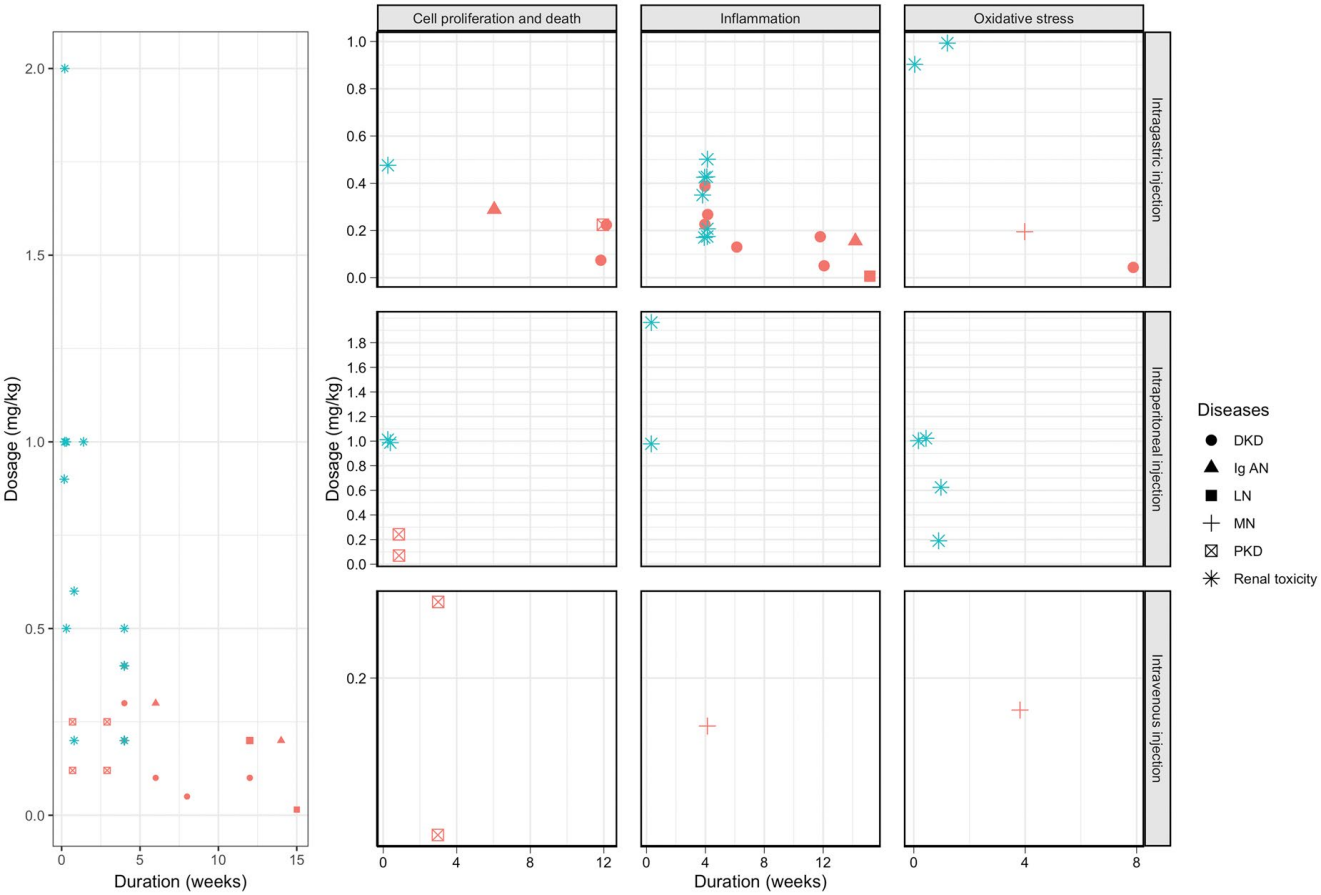
Distribution of triptolide dosage, duration, and biological effects in different disease states: *In vivo* experimental evidence. **Abb:** DKD, Diabetic Kidney Disease; Ig AN, Ig A Nephropathy; LN, lupus nephritis; MN, membranous nephropathy; PKD, polycystic kidney disease. Red dots represent the renal protection effect of Triptolide, and green dots represent the renal toxicity effect of Triptolide.

TP, a principal bioactive component of TwHF exhibiting potent efficacy in mitigating diverse kidney diseases while posing significant nephrotoxic risks. This review synthesizes evidence from *in vitro* and *in vivo* studies to elucidate its mechanisms of action, contextualizes its therapeutic-toxic balance, and highlights strategies to optimize its utility. Based on the distribution of data points in the charts (Figure 4), the therapeutic dose and toxic dose of TP are closely related to a narrow safe and effective range, implying that strict control of dosage and administration duration is imperative in applications. Taking DKD as an example within a single disease group, within the low-dose range (200–400 μg/kg), the distribution intervals on the vertical axis for data points representing therapeutic efficacy (indicators related to improved renal function) and toxicity (indicators associated with elevated inflammatory factors and enhanced oxidative stress) are similar. When the dosage increases to above 500 μg/kg, data points related to toxicity indicators such as inflammation and oxidative stress rise significantly, while no data points reflecting the therapeutic effects of TP appear within this dosage range. This phenomenon indicates that the toxic and therapeutic doses of TP are not uniformly distributed but exhibit certain clustering and dispersion characteristics, further emphasizing the importance of accurately determining the dosage during treatment. From a temporal perspective, analysis of the charts reveals partial overlap between data points of effective doses and toxic doses during short-term administration (2–4 weeks). However, TP treatment for most kidney diseases often requires long-term administration (4–12 weeks), and *in vivo* studies on the chronic toxicity of TP are lacking. This research gap hinders a comprehensive understanding of changes in the dose-toxicity relationship of TP during long-term administration, potentially affecting the accurate evaluation of its safety and efficacy. Thus, the dosage intervals for effective TP-induced therapy and toxicity overlap and clearly differ. Under the same administration duration, strict control of the TP dosage is crucial. To gain a deeper understanding of the safety and efficacy of TP in applications, it is necessary to conduct further preclinical studies on the long-term and chronic toxicity of TP in the future. Such studies will fill the current research gaps and provide a more solid theoretical basis for rational drug use.

TP exerts its renoprotective effects through multicellular and pathway-specific regulation. In RTECs, it affects abnormal proliferation in ADPKD and RCC by inhibiting JAK/STAT signaling, promoting calcium-dependent apoptosis, and sensitizing cells to TRAIL-mediated death. Under fibrotic conditions like DKD, TP attenuates EMT *via* TGF-β/Smad suppression and PTEN-mediated PI3K/Akt inhibition, while also counteracting MEX3C-driven PTEN ubiquitination to preserve tubular integrity.

In podocytes, TP mitigates proteinuria and FPE across models of MN, FSGS, and DKD. It achieves this by stabilizing slit diaphragm proteins (nephrin and podocin), inhibiting ROS-driven MAPK activation, and downregulating proapoptotic GADD45B expression *via* p53 suppression. Mesangial cell dysfunction, a hallmark of IgAN and DKD, is ameliorated through the modulation of autophagy by TP *via* CARD9/p38 MAPK and ECM deposition *via* PDK1/Akt and miR-137/Notch1 pathways. Additionally, the immunosuppressive properties of TP *via* JAK/STAT1 and NF-κB inhibition—alleviate LN and transplant rejection by reducing immune cell infiltration and cytokine release.

Despite these benefits, the narrow therapeutic window of TPis constrained by its selective toxicity to RTECs. At supratherapeutic doses or with prolonged exposure, TP disrupts tubular structure and function by upregulating OCT2 expression to enhance renal accumulation, impairing the expression of tight junction proteins (ZO-1 and JAM-1) and glucose transporters (SGLT1/2), and triggering oxidative stress *via* NRF2-BACH1 imbalance. This oxidative burst activates cGAS/STING signaling, propagating inflammation and apoptosis. Metabolically, TP induces phase I/II biotransformation imbalances, depletes GSH, and dysregulates purine metabolism—*via* ADA upregulation—leading to adenosine depletion, ROS overproduction, and TLR/NF-κB-mediated injury. These interconnected pathways form a vicious cycle: TP-induced stress enhances its own renal uptake, exacerbating toxicity.

Innovative approaches are emerging to harness the benefits of TP while minimizing harm, such as targeted delivery systems that enhance RTECs-specific accumulation, structural derivatives (LLDT-8, PG490-88, and Min) optimize pharmacokinetics and combination therapies modulate TP’s half-life and distribution of TP, lowering peak plasma concentrations without compromising renal efficacy. Other potential combinations warrant exploration, such as pairing TP with antioxidants to counteract TP-induced oxidative stress (Nie et al. 2024), or with inhibitors of organic cation transporter 2 (OCT2) to reduce RTECs-specific uptake of TP. These strategies could further enhance the therapeutic index of TP by targeting distinct mechanisms of toxicity (oxidative stress and cellular accumulation) while preserving its anti-inflammatory and anti-fibrotic efficacy.

Gaps in critical knowledge remain, regarding the cell-type-specific molecular targets of TP. Advanced techniques such as thermal proteome profiling and biotinylated probes can be used to identify direct interactors to clarify their dual effects (Zhao et al. 2022b; Chen et al. 2023). Additionally, the ‘dose–time–cell type’ dynamics—such as low-dose, short-term administration for podocyte protection versus pulsed dosing for PKD/RCC—warrant systematic investigation to refine therapeutic protocols. In summary, the therapeutic potential of TP in renal disorders is undeniable, but its translation requires precision. By integrating targeted delivery, structural modification, and mechanistic insights into its context-dependent effects, TP may be a promising therapy for kidney diseases.

## Data Availability

Data sharing is not applicable to this article.
